# Stepped-care cognitive behaviour therapy program for treating cancer-related fatigue: protocol for a feasibility study

**DOI:** 10.1186/s40814-022-01062-8

**Published:** 2022-05-27

**Authors:** Lauren K. Williams, Maria Ftanou, Elizabeth J. Pearson

**Affiliations:** 1 Department of Health Services Research, Peter MacCallum Cancer Centre, Level 4, 535 Elizabeth Street, Melbourne, Victoria 3000 Australia; 2 Department of Psychology, Peter MacCallum Cancer Centre, 305 Grattan Street, Melbourne, Victoria 3000 Australia

**Keywords:** Cancer, Fatigue, CBT, Psychological, Survivor, Stepped-care, Cognitive behaviour therapy

## Abstract

**Background:**

Cancer-related fatigue (CRF) is a commonly experienced and often debilitating side effect of cancer treatment that can persist for years after treatment completion. The benefits of cognitive behaviour therapy (CBT) for CRF are well established; however, these interventions are typically not included in standard clinical care. Traditional CBT is resource-intensive, limiting implementation in hospital settings. Stepped-care approaches can offer benefits to more people, using the same personnel as traditional models.

**Method/design:**

This is a single-arm feasibility study. Fifty people with a cancer diagnosis, at least 12 weeks post-treatment or on long-term maintenance treatment, with persistent CRF that is affecting daily activities, will enrol in a stepped-care CBT program. Intervention: The stepped-care program involves two steps. Step 1: All participants begin with a 5-week supported self-management CBT progam targeting fatigue. Step 2: If fatigue remains severe or has changed less than the minimal clinically important difference on the fatigue measure after step 1, participants will be offered four sessions of therapist-directed group CBT. Measures: Participants will complete questionnaires at baseline and 6 and 10 weeks. The primary outcome is feasibility of the REFRESH program. The implementation evaluation comprises acceptability, satisfaction, appropriateness, and feasibility of the study intervention, along with administrative data including cost, processes, procedures and implementation. Secondary outcomes are changes in fatigue, quality of life and self-efficacy.

**Conclusion:**

The REFRESH program will be the first stepped-care CBT intervention for persistent CRF in Australia. Assessing feasibility of REFRESH is an important first step to establishing future implementation and efficacy.

## Background

Cancer-related fatigue (CRF) refers to the distressing and persistent subjective sense of physical, emotional and/or cognitive exhaustion related to cancer and/or cancer treatment [1]. Further, CRF is not proportional to recent activity and is not alleviated with sleep or rest [2] Approximately half of people receiving cancer treatment report moderate to severe CRF, and it can be debilitating years after treatment completion for up to one in three survivors [3]. Fatigue significantly interferes with everyday tasks, employment, physical and social activities. It further inhibits functional recovery and treatment adherence, with additional adverse impact on functional cognition (e.g. attention and concentration) and emotional wellbeing, including increased depression symptoms and lower quality of life [4, 5]. Whilst there is some evidence that fatigue has little impact on task performance in advanced cancer [6], the distressing nature and consequences of fatigue symptoms can be significant for many months or years following cancer treatment [7, 8].

Medications for CRF are not recommended except to relieve suffering in advanced disease [9] and non-pharmacologic interventions are therefore recommended to treat CRF [10]. Cognitive behaviour therapy (CBT) is an evidence based psychological treatment used for a range of psychological symptoms [11] and is the internationally recommended first line of treatment for persistent CRF [10]. The goal of CBT is to change unhelpful ways of thinking and modify learned patterns of unhelpful behaviour to reduce unpleasant emotions and physical distress.

The causal assumption for effectiveness of CBT for CRF relates to addressing the psychological and lifestyle factors that maintain fatigue [12, 13]. Physical inactivity or over-activity, worry and rumination, mood disturbance, sleep problems, pain, inadequate nutrition and low self-efficacy have all been identified as maintaining factors for CRF [10, 14]. These maintaining factors of CRF are bi-directional and create a vicious cycle for individuals. Cognitive and behavioural factors associated with increased fatigue and poorer functioning include all-or-nothing thinking, catastrophizing and avoidance behaviour [15]. The goal of CBT for persistent CRF is to target unhelpful behaviours, thoughts and emotions associated with CRF maintaining factors in order to reduce fatigue severity and fatigue-related disability [16].

The effectiveness of CBT for CRF has been established in a small number of studies across the Netherlands, Germany and UK [12, 16, 17]. In a recent systematic review of the effectiveness of psychological interventions for fatigue in cancer survivors, 12 of the 33 randomised controlled trials reported on the effects of CBT on CRF [18]. Five of these 12 studies compared CBT with treatment as usual, all reporting a significant decline over time in fatigue for the intervention group. The remaining seven studies in this review combined CBT with, or compared CBT to, another intervention, typically physical activity [18]. Whilst these results are promising, the CBT programs varied considerably in content and delivery, and only two targeted fatigue. Several programs included a high treatment dose of up to 26 face-to-face or online sessions with a psychologist, and others utilised web-based methods.

Resource-intensive programs for CRF are simply not feasible and sustainable, where healthcare manpower or expertise are limited. Additionally, a program must have demand, be acceptable, affordable, accessible and show promise for efficacy in order to be feasible longer term [19] The feasibility of embedding a CBT program for fatigue into “real world” clinical settings (outside of the research context) that considers these program priorities has not been established in Australian cancer services.

One approach to enhancing feasibility of CBT for CRF, particularly in a resource restricted environment, is to use a stepped-care intervention that can cater for individuals with differing needs. An example of stepped-care is to provide low-intensity treatments (e.g. self-help resources) as a starting point, with a ‘stepped up’ pathway to specialist services as clinically required. Whilst therapist-directed CBT is gold standard delivery, self-directed CBT is also effective [20, 21], and therefore utilising a stepped-care model of CBT can potentially benefit more patients with the same personnel as traditional delivery.

**Fig. 1:**
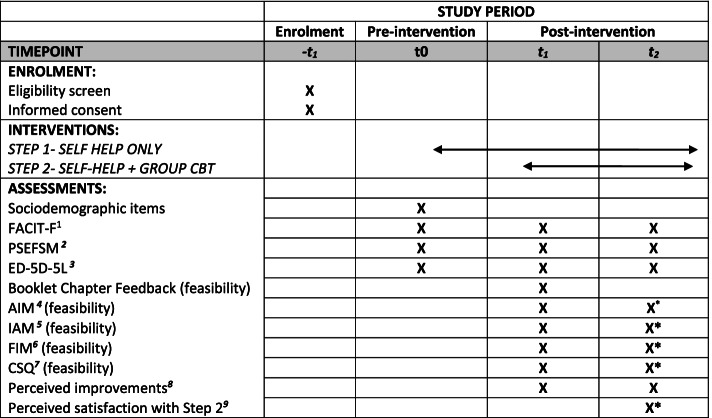
SPIRIT: overview of REFRESH. Superscript digit one (1) indicates the following: the Functional Assessment of Chronic Illness Therapy—Fatigue; superscript digit two (2) indicates the following: Perceived Self Efficacy for Fatigue Self-Management; superscript digit three (3) indicates the following: European Quality of Life 5 Dimension 5 Level; superscript digit four (4) indicates the following: Acceptability of Intervention Measure; superscript digit five (5) indicates the following: Intervention Appropriateness Measure; superscript digit six (6) indicates the following: Feasibility of Intervention Measure; superscript digit seven (7) indicates the following: Client Satisfaction Questionnaire; superscript digit eight (8) indicates the following: perceived improvements to personal, social and lifestyle factors (self-devised); superscript digit nine (9) indicates the following: perceived satisfaction with step 2 (self-devised). Asterisk (*) indicates the following: these measures will only be completed in the t2 questionnaire by those who have completed STEP 1 and STEP 2 of the intervention

This paper describes a research protocol to investigate the feasibility of stepped-care CBT for individuals experiencing persistent CRF, entitled ‘REFRESH’ (ACTRN: ACTRN12622000420741). This is a single-arm exploratory study design. Standard Protocol Items: Recommendations for Interventional Trials (SPIRIT) guidelines were used to guide the reporting of the current feasibility study (see Fig. 1). The primary aim of the REFRESH study is to examine the acceptability, satisfaction, appropriateness and feasibility of an evidence-informed stepped-care CBT intervention for adults with persistent fatigue after cancer treatment. A secondary aim is to explore whether participation in the stepped-care intervention shows promise for improvements in fatigue, quality of life and self-efficacy.

## Methods

### Participants

Fifty adults with a cancer diagnosis who have completed treatment at Peter MacCallum Cancer Centre in Melbourne, Australia, at least 12 weeks prior to study enrolment, or are on long-term maintenance treatment, who have moderate to severe CRF will be recruited to REFRESH.

### Participant inclusion criteria

Each participant must meet the following criteria to participate in the study:

- Aged 18 years or older at the time of recruitment, able to speak and read English
- Reports moderate to severe persistent fatigue based on study screening questions
- Completed primary treatment for any cancer at least 3 months prior
- OR has been on long term maintenance treatment (e.g. single agent immunotherapy or targeted therapy) for melanoma or a blood cancer for at least 3 months with partial or complete tumour response
- OR is diagnosed with a chronic blood cancer but below treatment threshold (no active treatment or ‘watch and wait’)

### Participant exclusion criteria

Participants will be excluded from the study if the clinical screening interview identifies:

- A likely significant sleep disorder and/or
- Current psychosis, significant psychological distress or risk of self-harm
- Fatigue is not *persistent* (i.e. duration less than 4 weeks)

### Participant recruitment and consent

Participants will be recruited from outpatient specialist clinics at Peter MacCallum Cancer Centre. Tumour stream nurses, medical or allied health professionals will identify patients, during routine clinical care, who are reporting fatigue symptoms. Additional recruitment measures will include promotional postcards in waiting areas, study advertisements in newsletters, emails to medical and allied health staff and presentations at staff meetings. The health professional will then notify the research team. A researcher will telephone the patient to discuss the study and, if the person indicates interest, will screen for eligibility. The eligibility screen includes a series of questions based on Canadian guidelines for assessing CRF [10]. The eligibility screening questions are (1) a self-report score of 4 or more to the question “On a 0 to 10 scale where 0 means no fatigue and 10 means the worst fatigue imaginable, how would you rate your fatigue at its worst over the past 3 days?”; (2) moderate to severe symptoms of fatigue assessed using qualitative descriptors adapted from Canadian fatigue guideline [10] by endorsing any item indicating moderate to severe fatigue (e.g. “fatigue is noticeable and upsetting” (moderate fatigue) or “exercise does not seem possible” (severe fatigue); and (3) endorsing ‘yes’ to the question “Has fatigue been affecting your day to day life for one month or more?”.

Those meeting full eligibility criteria will be offered follow up care in the REFRESH study. People with mild fatigue, who are not eligible for the research study, and individuals who are eligible but decline participation will be directed to online CRF resources to prevent exacerbation of fatigue symptoms.

### Study procedures

#### Pre-intervention assessments (T0)

**Fig. 2:**
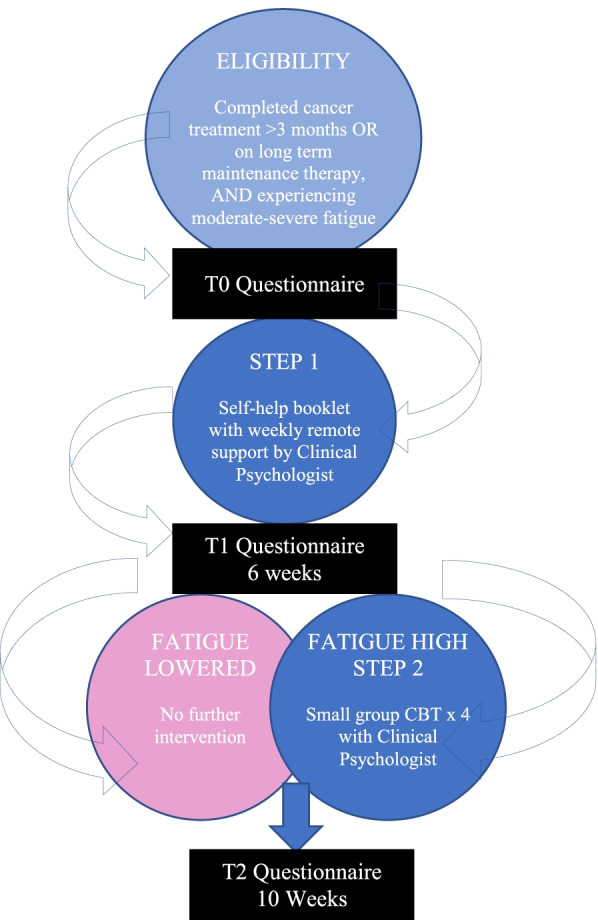
Model of REFRESH: finding new energy after cancer stepped care

After signing consent, participants will complete the baseline questionnaire (T0), online or on paper. Sections include demographics, fatigue, quality of life and self-efficacy. See 'participant psychosocial measures' section below. After submitting the T0 questionnaire, all participants will receive the REFRESH self-help booklet in hard copy (given in person or sent in the mail) and commence STEP 1. Figure 2 depicts study procedures.

#### STEP 1: Supported self-management and REFRESH self-help booklet

The REFRESH self-help booklet was developed by experienced health professionals and researchers based on existing literature. A multidisciplinary panel of experts, consumers, health literacy specialists and professional graphic designers contributed to refine and produce the REFRESH booklet. The self-help booklet is structured into five chapters (see Table 1). Participants will be encouraged to read and complete the activities of one chapter per week, for 5 weeks. Each week participants will be contacted by a study psychologist both via email (weeks 1, 3 and 4) and via telephone (weeks 2 and 5). The purpose of these weekly contacts is to discuss and support participation, answer questions and troubleshoot problems. If participants miss a chapter, they will be encouraged to proceed with the next chapter and take note of any sections they feel are relevant to them from the preceding chapter. Each week, they will also complete some feedback questions pertaining to the chapter completed/attempted.

**Table 1:** REFRESH session goals and objectives

STEP 1—chapter #	STEP 2-session #	Session topic	Session goals and objectives
1	n/a	Understanding fatigue	To provide information and psycho-education about cancer-related fatigue including medical and psychosocial causes of CRF, and explanation of CBT and its role in CRF. Introduction to an activity diary for tracking fatigue
2	**1**	Understanding feelings	To understand the impact of emotions on fatigue and strategies to increase pleasant emotions. Introduction to relaxation strategies (breathing, muscle relaxation and mindfulness)
3	**2**	Helpful behaviours	To understand the role of behaviours in impacting fatigue. Mood monitoring, increasing physical activity, pacing and scheduling pleasant and mastery activities for the week ahead as part of behavioural activation
4	**3**	Adaptive thinking	To understand the role of cognitions in influencing emotions, behaviour and fatigue. Identifying unhelpful thoughts and ‘thinking traps’, cognitive restructuring via use of a thought diary. Introduction to worry postponement strategy and coping statements
5	**4**	Moving on	Summarise skills learnt and identify which are helpful and can be implemented ongoing. Assistance with developing a coping card if this hasn’t been done prior. Assistance with identifying additional supports. Assistance with accessing further professional supports if needed

As a contingency, participants who are too distressed or unable to engage with the self-help program within two weeks after commencing STEP 1 can be fast tracked to STEP 2 (see below).

#### Time 1 assessments (T1)

After 5 weeks, participants will complete a follow-up questionnaire. The T1 questionnaire includes fatigue, quality of life and self-efficacy questions (repeated T0 measures), in addition to feasibility and satisfaction measures (see “study measures” below).

#### Eligibility for STEP 2

Eligibility for STEP 2 is based on a participant’s change in fatigue scores following STEP 1. Fatigue will be measured using the Functional Assessment of Chronic Illness Therapy—fatigue subscale (FACIT-F) scores [22]. Participants with a T1 FACIT-F score greater than 34 or at least 10 points greater than T0 will be encouraged to continue their strategies without further CBT intervention. Participants with a FACIT-F score on the T1 questionnaire below 34 (severe) or less than 10 points greater than T0 will be considered for STEP 2 in conjunction with a psychologist’s clinical judgement of further support needs.

#### STEP 2: Individual or group CBT delivered by a clinical psychologist

STEP 2 participants will be offered small group CBT therapy (maximum *n* = 5 per group). Therapy will comprise up to four 50-min sessions with a clinical psychologist either face-to-face or via telehealth. Clinical psychologists in Australia have a minimum of 6 years university-level training with an additional 2 years of supervised practice. The mode of delivery (i.e. telehealth vs face-to-face) will be decided based on COVID-19 restrictions (e.g. face-to-face groups may be prohibited with statewide laws) and participant circumstances (e.g. location of participant is rural and lengthy travel time would prohibit participation). Individual sessions will be offered to those where group therapy is not appropriate (e.g. due to extensive work or parenting commitments). Sessions will be based on content in chapters 2–5 of the self-help booklet (see Table 1) and tailored to the group based on their particular concerns and areas of need. A therapist manual has been developed to assist facilitation and delivery consistency. Face-to-face sessions will be held in a clinic room at Peter MacCallum Cancer Centre.

#### Time 2 assessments (T2)

At 10 weeks post T0 (for STEP 1 only completers) or at the completion of STEP 2, all participants will complete a final T2 questionnaire. Completion of the T2 questionnaire marks the end of participation in the REFRESH study.

### Outcome measures

Measures are summarised as (1) implementation outcome measures and (2) participant psychosocial measures.

### Implementation outcome measures

The implementation evaluation plan is guided by the Medical Research Council (MRC) framework for conducting and reporting process evaluations [23]. This means that rather than focussing on intervention effects (which assume adequate sample size, randomisation etc.), the focus is on understanding *how* interventions work in practice and the mechanisms for behaviour change. Therefore, there is more emphasis on factors such as whether participants were able to work through the self-help booklet, rather than focussing solely on changes to outcome measures. This approach assists evaluators to decide aspects of the intervention or its context to prioritise for further investigation or clinical implementation. The implementation evaluation includes acceptability, satisfaction, appropriateness, feasibility of the stepped-care intervention, recruitment, retention, adherence, cost and fidelity. Table 2 summarises the evaluation processes and measures.

**Table 2:** REFRESH evaluation plan and measures

Implementation outcomes	Definition	Rationale	Method of evaluation
**Acceptability and Satisfaction**	The perception amongst users that the program is palatable and satisfactory [24]	Users consider the program acceptable	**□ The Acceptability of Intervention Measure (AIM)**^a^ **□ Client Satisfaction Questionnaire (CSQ)**^b^ **□** Recruitment and withdrawal records **□** Open-ended study-devised questionnaire items (e.g. perceived barriers and/or helpfulness of chapters)
**Appropriateness**	The perceived fit and relevance of the program to users [24], indicated by proportion of people willing to try and complete the program	Consumers perceive it is worth trying and completing the program	**□ The Intervention Appropriateness Measure (IAM)**^a^ **□** Retention (proportion of enrolled participants who completed follow-up assessments; proportion of participants who completed each module of step 1; proportion of participants who completed step 2) **□** Adherence to step 1 and step 2 (percentage of sessions attended, reasons for not completing sessions, number of days overdue for completed questionnaires).
**Feasibility of materials and CBT group attendance**	The extent to which stepped care approach to CBT can be used for cancer fatigue	A majority of participants complete step 1 in 6 weeks and/or step 2 in 4 sessions	**□ The Feasibility of Intervention Measure (FIM)**^a^ **□** Open-ended study-devised questionnaire items (e.g. number of chapters of the self-management booklet attempted/completed)
**Cost**	The resource cost of delivering the program per user	This self-help CBT program is expected to provide therapy at a lower cost compared to face to face, enabling more users to benefit	**□** Booklet cost and records of therapist/administration time per person enrolled **□** Proportion of participants requiring step 2
**Fidelity**	The degree to which the program was implemented as intended [24]	The adherence and deviations to program contacts and stepped care protocol	**□** Field notes maintained by researchers **□** Session checklist after each STEP 2 session to ensure all topics covered

^a^The AIM, IAM and FIM are 4-item (each) validated measures with items rated on a 5-point Likert scale from ’completely disagree’ to ‘completely agree’ with higher scores indicating greater intervention acceptability, appropriateness and feasibility respectively [25]

^b^The CSQ [26] is a well validated 8-item measure of the quality of the intervention, the extent to which the program met participant’s needs, perceived increases in skills and whether participants would recommend the program to others. Total scores range from 8 to 56, with higher scores indicating greater satisfaction

### Participant details and psychosocial measures

#### Patient demographic and medical data

Sociodemographic characteristics will be collected on enrolment to gather descriptive information about which participants were drawn to and retained in the study: diagnosis and treatment details, age, sex, country of birth, language spoken, education, marital status and employment status.

#### FACIT-Fatigue (Functional Assessment of Chronic Illness Therapy—Fatigue subscale)

The FACIT-F is a valid measure of the severity of cancer-related fatigue, with a recall period of 7 days [22]. FACIT-F is a 13-item scale, with items rated on a 5-point Likert scale from 0 (not at all) to 4 (very much). Score range is 0–52, with the population mean of 43 [27]. Lower scores on the FACIT-F indicate greater fatigue and impairment. A FACIT-F score below 34 is considered to be severe [28] and a 10-point improvement a clinically important difference [29].

#### Perceived self-efficacy for fatigue self-management scale (PSEFSM)

The PSEFSM is a valid 6-item instrument developed to measure self-efficacy in CRF management [30]. An 11-point scale (0–10, 10 = very certain) measures perceived ability to self-manage fatigue. Scores of the six items are averaged and final score ranges from 0 to 10 with 10 being highest perceived self-efficacy.

#### EuroQol 5 Dimension 5 Level Scale (EQ-5D-5L)

The EQ-5D-5L is a 5-item measure of health-related quality of life (HRQoL) [31]. One item is used to rate each dimension of mobility, self-care, usual activities, pain/discomfort and anxiety/depression on a Likert scale. Response options are as follows: no problems (0), slight problems (1), moderate problems (2), severe problems (3) and extreme problems (4). A final question asks the participant to rate their health from 0 (worst) to 100 (best) on a visual analogue scale. Scores for the five dimensions can be added to describe the patient’s health state with a possible range 0–20, with higher scores indicating worse HRQoL.

#### Perceived changes to personal, social and lifestyle factors

A study-devised questionnaire assesses perceived changes in personal, social and lifestyle factors related to fatigue that are targeted in the REFRESH program. These are sleep, mood, anxiety/worry, energy, exercise levels, social activity and engagement in hobbies. Response options for each item are follows: ‘Better’, ‘Same’ or ‘Worse’ (since starting the program). If participants select ‘Better’ on any given item, they are further prompted to record which stage of the booklet or program they believe the change/benefit for that domain was noticed.

### Data analysis

The target sample of 50 participants is based on available funding, resources (e.g. clinician time to deliver STEP 2) and study duration of 12 months. Whilst this sample size is in line with pilot and feasibility trials reported elsewhere [32], there is yet to be a clear consensus as to appropriate sample size for feasibility studies [33].

Analyses will include all available data and will be performed in R (reference index version 3.6.1 or higher) [34]. Descriptive statistics will be used to summarise demographic and clinical characteristics of all study participants. These will include counts and percentages for nominal and crude-scale ordinal (< 10 levels) valued variables and means and standard deviations or medians and interquartile ranges, as appropriate, for fine-scale (≥10 levels) ordinal and continuous valued variables.

The main implementation outcomes are acceptability, satisfaction, appropriateness and intervention feasibility of the stepped-care CBT intervention. These will be summarised using descriptive statistics. Recruitment data will be summarised using a rate and 95% CI using the Poisson distribution. Adherence and retention data will be summarised using a proportion and 95% CI; this will be estimated using the Wilson method.

Changes from baseline at follow-up assessments for fatigue (FACIT-F), self-efficacy (PSEFSM) and quality of life (Ed-5D-5L) will be analysed descriptively (means and standard deviations). This will be done separately for participants who undertake STEP 1 only and for those who participate in STEP 1 and STEP 2. Effect size estimates (i.e. standardised measures of change from baseline; in this case, mean change divided by the baseline standard deviation), as described by Kazis, Anderson and Meenen [35] will be used to characterise the size of observed differences.

Free text items from participant questionnaires will be summarised using content analysis, whereby the content of free responses will be coded and grouped, where applicable. Since the free text items mostly accompany pre-determined response options, it is not envisaged that all participants will respond to free-text items.

## Discussion

The REFRESH study is the first to explore the feasibility of a stepped-care approach to persistent CRF amongst individuals who have completed cancer treatment or are on long term maintenance treatment. With a pragmatic study design, it aligns with common clinical practice. This may facilitate translation of the stepped-care intervention into practice. Although not powered to determine effectiveness, if at least half of the participants achieve a clinically meaningful improvement using supported self-management (STEP 1) alone, it will have the potential to reach many people suffering with CRF. Each person needing STEP 2 will require an additional 4 h of therapist time and some administrative time. This increases costs per participant and service capacity to deliver. Therefore, STEP 2 will be offered pragmatically in small group formats to manage demand and adhere to project timelines, as would occur in real world practice.

If feasible, a stepped-care approach could enable many survivors to access CBT for post-cancer fatigue.

## Data Availability

Please contact LW for REFRESH program details. The final dataset will remain the property of Peter MacCallum Cancer Centre.

## Abbreviations

CBT: Cognitive behaviour therapy
CRF: Cancer-related fatigue
